# Genetic Testing of Neurodevelopmental Disorders in Israel

**DOI:** 10.1001/jamanetworkopen.2025.27464

**Published:** 2025-08-19

**Authors:** Dalit May, Ruth Barshir, Moni Shahar, Adam J. Rose, Dorit Shmueli

**Affiliations:** 1 Clalit Health Services, Jerusalem, Israel; 2 The Fuld Family Medical Genetics Institute, Shaare Zedek Medical Center, Jerusalem, Israel; 3 The Eisenberg Research and Development Authority, Shaare Zedek Medical Center, Jerusalem, Israel; 4 The Tel Aviv University Center for AI and Data Science, Tel Aviv, Israel; 5 Hebrew University School of Public Health, Jerusalem, Israel

## Abstract

**Importance:**

Genetic testing is the criterion standard for diagnosing neurodevelopmental disorders (NDDs), with chromosomal microarray analysis (CMA) used as a first-line test for autism, intellectual disability, or global developmental delay. Despite advancements in genetic testing technologies and integration into health care systems, data on clinical use remain limited.

**Objective:**

To evaluate genetic counseling and testing rates in patients with major NDDs and these individuals’ clinical and sociodemographic characteristics.

**Design, Setting, and Participants:**

This longitudinal, retrospective, population-based cohort study analyzed electronic health records of individuals born between 2000 and 2020 and insured by Clalit Health Services, the largest health maintenance organization in Israel. Follow-up extended through December 6, 2023. Patients diagnosed with autism spectrum disorder, intellectual disability or global developmental delay, epilepsy, or cerebral palsy (major NDDs) were included.

**Exposure:**

Neurodevelopmental disorders.

**Main Outcomes and Measures:**

The outcome was the rate of genetic counseling, CMA testing, and NDD diagnosis measured using descriptive statistics.

**Results:**

Of 2 406 763 individuals born in Israel between 2000 and 2020, 25 403 (1.06%; mean [SD] age at December 6, 2023, 11.9 [4.3] years; 68.7% male) were diagnosed with a major NDD. The cohort was predominantly of middle socioeconomic status (56.5%), and autism was the most common diagnosis (40.6%). Among 18 709 children indicated for CMA (ie, those with autism, intellectual disability or global developmental delay, or multiple diagnoses), 7233 (38.7%) received genetic counseling, and 4592 (24.5%) underwent testing (63.5% of those counseled). Genetic testing rates were higher in children with multiple co-occurring NDDs (1478 of 4005 [36.9%]) compared with those with autism alone (2189 of 10 311 [21.2%]). Genetic counseling rates were lowest for cerebral palsy and epilepsy as guidelines were less established. Genetic evaluation rates increased with more recent birth cohorts. While evaluation rates were similar across subpopulations for children with a diagnosis, initial autism diagnosis rates were 54% to 83% lower in lower socioeconomic status and minority populations, limiting access to counseling and testing.

**Conclusions and Relevance:**

A key finding of this cohort study was that more than one-third of patients who received genetic counseling did not undergo testing. Furthermore, low socioeconomic status and minority populations experienced drastic underdiagnosis of autism. These findings underscore the need for national initiatives to improve awareness and access to counseling and testing for all major NDDs and the recognition of autism in minority groups.

## Key Points

**Question:**

What are the rates of genetic counseling and testing among children with major neurodevelopmental disorders in Israel, and do these differ across subpopulations?

**Findings:**

In this cohort study of 2 406 763 children, 1.06% were diagnosed with major neurodevelopmental disorders, with a 54% to 80% underdiagnosis of autism among those of low socioeconomic status and minority groups. Overall, 38.7% of patients indicated for chromosomal microarray analysis received genetic counseling, and 24.5% underwent testing; rates were similar across subpopulations.

**Meaning:**

The overall underuse of genetic evaluation for neurodevelopmental disorders and the underdiagnosis of autism in certain subpopulations highlight the need for awareness and equity.

## Teaser

This cohort study evaluates genetic counseling and testing rates in Israel among children with major neurodevelopmental disorders and their clinical and sociodemographic characteristics.

## Introduction

Neurodevelopmental disorders (NDDs) encompass a range of conditions resulting from abnormal brain and central nervous system development, typically manifesting in early childhood and affecting cognitive, motor, and behavioral functions.^1,2,3^ As genetic testing has become more accurate and affordable, it is now recommended as the standard of care for children diagnosed with major NDDs, including autism spectrum disorder (hereafter referred to as autism) and intellectual disability or global developmental delay (ID/GDD).^4,5,6,7,8^ Genetic evaluation also extends to other NDDs, such as cerebral palsy^9,10,11,12,13^ and epilepsy.^14,15,16,17^ Medical organizations, including the American Academy of Child and Adolescent Psychiatry and the American College of Medical Genetics and Genomics, recommend genetic testing for NDDs, particularly autism.^18,19^

A genetic diagnosis enhances understanding, enables early interventions, and improves management strategies, benefiting developmental and medical outcomes.^20,21^ Testing may expedite the diagnostic process for quicker access to definitive diagnoses and provision of critical information for family planning regarding recurrence risks in future pregnancies.^4^

Genetic evaluation typically involves genetic counseling performed by board-certified clinical geneticists followed by laboratory testing. Chromosomal microarray analysis (CMA) is a high-resolution genetic testing technique that detects submicroscopic chromosomal abnormalities, such as copy number variations, associated with NDDs.^22,23,24^ Introduced into clinical practice around 2010, CMA has been covered by Israel’s national health care system since 2013, primarily for individuals with autism, ID/GDD, or multiple NDDs.

Next-generation sequencing technologies, including exome sequencing, which targets all protein-coding regions and genome sequencing, covering both coding and noncoding regions, represent key advancements in clinical genetics, and their diagnostic yield has been shown to surpass that of CMA.^25,26,27,28,29,30^ However, their clinical use is yet to be fully incorporated into Israel’s national health program, and comprehensive data are currently lacking. Meanwhile, CMA has remained the first-line genetic test for major NDDs, serving as the most reliable indicator of genetic testing use.

Few studies have examined genetic testing use and have mainly focused on autism, showing low but increasing rates. However, small cohorts, limited population representation, and a limited scope on autism have overlooked other NDDs with potentially higher diagnostic yields.^31,32^ In Israel, while testing is covered by national health insurance, access is hindered by long wait times, bureaucracy, and limited awareness among families and primary care physicians.^2,8^

This population-based study aims to evaluate the use of genetic counseling and testing among children diagnosed with major NDDs in Israel, considering variations across different disorders, age groups, socioeconomic status (SES), social and ethnic groups, and sex. In addition, this study evaluates the rates of NDD diagnosis in the population.

## Methods

### Study Population

In this cohort study, we followed up a cohort of children born between January 1, 2000, and December 31, 2020, from birth until December 6, 2023. The study included only children who were members of Clalit Health Services (CHS), the largest health management organization in Israel that covers approximately 52% of the Israeli population and maintains a comprehensive digital database and registry. The standard practice of the CHS is to refer children suspected of having NDDs to a child neurologist for further evaluation. Diagnosis of NDDs made by neurologists were based on clinical presentation and supporting tests in accordance with clinical guidelines, including the *Diagnostic and Statistical Manual of Mental Disorders* (Fifth Edition), when relevant. This study was approved by the institutional review boards of Meir Medical Center and CHS. Obtaining informed consent from study participants was waived by the institutional review boards given the retrospective nature of the study and the anonymity of the data. This study followed the Strengthening the Reporting of Observational Studies in Epidemiology (STROBE) reporting guideline.

### Data Extraction

The study data were extracted on December 6, 2023, from the CHS database, which contains comprehensive clinical information on diagnoses, hospitalizations, procedures, visits to outpatient clinics, and consultations with specialist physicians. Sociodemographic data, such as date of birth, sex, geographic region of residence, population sector (general Jewish, Arab, or Ultra-Orthodox Jewish), and SES score (low, middle, or high) were extracted for each enrolled patient. Socioeconomic status scores in the CHS database were assigned based on the patient’s address, using a standardized neighborhood-level index developed by the Israeli Central Bureau of Statistics.^33^ Child development services, hearing tests, genetic counseling, and CMA testing were identified through records of procedures performed in CHS hospitals, visits to relevant outpatient clinics, and consultations with specialist physicians. For genetic counseling and CMA testing, additional records were obtained through proof of payment to individual practitioners and external institutions to ensure complete capture of such data.

### Study Group Ascertainment

Diagnoses of NDD were grouped into 7 categories (eTable 1 in Supplement 1), which were further classified as either major NDDs, including autism, ID/GDD, epilepsy, and cerebral palsy, or minor NDDs, including attention-deficit/hyperactivity disorder, delayed motor milestones, and mixed developmental disorder. The initial NDD cohort was based on diagnosis records through December 31, 2020. An updated data extraction on December 6, 2023, captured the most recent services received by the cohort.

### Statistical Analysis

Data analysis was performed using Python, version 3.9.7 (Python Software Foundation). Statistical significance was assessed using *z* tests conducted using the Python library statsmodels.stats.proportion.proportions_ztest. Proportions and corresponding 95% CIs were calculated using the Python scipy.stats.binomtest module. When multiple comparisons were performed, *P* values were adjusted using the Benjamini-Hochberg procedure to control the false discovery rate. Results were deemed statistically significant at *P* < .001.

## Results

### Prevalence of NDDs

**Figure 1.**
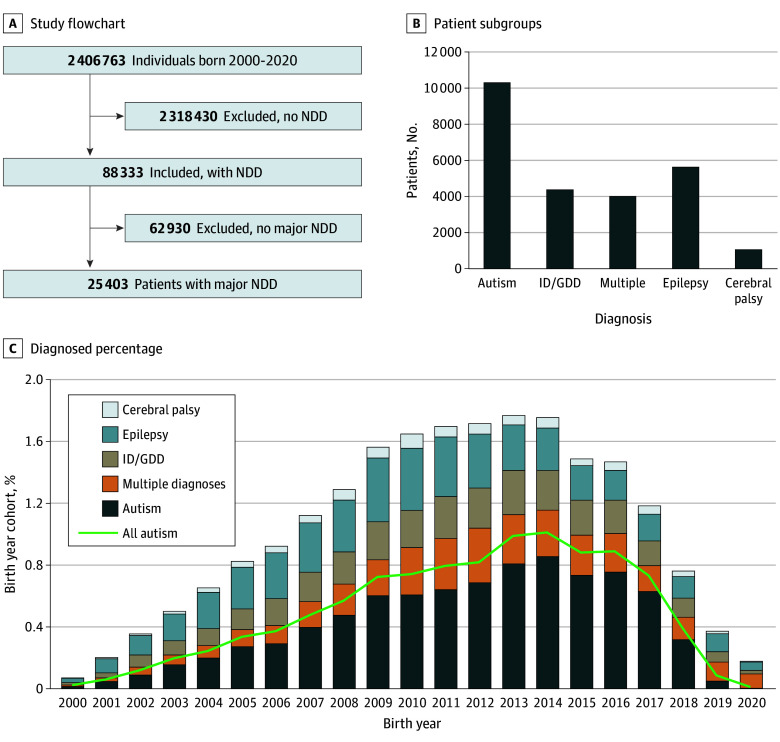
Description of the Study Cohort ID/GDD indicates intellectual disability or global developmental delay; NDD, neurodevelopmental disorder.

In this cohort study of 2 406 763 individuals insured by CHS and born between 2000 and 2020, we identified 88 333 patients diagnosed with any NDD before the end of 2020 and before age 10 years, representing 3.7% of the total population (Figure 1A), consistent with reported prevalence.^1^ To ensure the relevance for diagnoses for which genetic evaluation was warranted, we focused on the subset of patients diagnosed with major NDDs. Patients diagnosed with mild NDDs, such as attention-deficit/hyperactivity disorder, for which genetic evaluation may not be routinely recommended, were excluded (eTable 1 in Supplement 1). The major NDD cohort included 25 403 patients, representing 1.06% of the insured population. This cohort included 68.7% male patients and 31.3% female patients (Table), reflecting a male-to-female ratio of 2.2:1 consistent with expected sex distributions in NDDs.^34,35^ The mean (SD) age of this sample as of December 6, 2023, was 11.9 (4.3) years. The majority of the cohort were of middle SES (56.5%), and a considerable number of children were from Arab (23.6%) or Ultra-Orthodox Jewish (10.4%) communities compared with the general Jewish population (66.0%).

**Table.** Clinical, Socioeconomic, and Demographic Characteristics of the NDD Cohort (N = 25 403)

Characteristic	Patients, No. (%)		
	Total	Genetic counseling^a^	CMA testing^a^
Diagnosis			
Autism	10 311 (40.6)	3422 (33.2)	2189 (21.2)
ID/GDD	4393 (17.3)	1642 (37.4)	925 (21.1)
Multiple diagnoses	4005 (15.8)	2169 (54.2)	1478 (36.9)
Indicated group^b^	18 709 (73.6)	7233 (38.7)	4592 (24.5)
Epilepsy	5632 (22.2)	1385 (24.6)	542 (9.6)
Cerebral palsy	1062 (4.2)	261 (24.6)	131 (12.3)
Total	25 403 (100)	8879 (35.0)	5265 (20.7)
Sex			
Female	7948 (31.3)	2831 (35.6)	1561 (19.6)
Male	17 454 (68.7)	6048 (34.7)	3705 (21.2)
Socioeconomic status			
Low	5948 (23.4)	2169 (36.5)	1241 (20.9)
Middle	14 365 (56.5)	5004 (34.8)	3012 (21.0)
High	3654 (14.4)	1148 (31.4)	697 (19.1)
Unknown	1436 (5.7)	558 (38.9)	316 (22.0)
Ethnic population			
General	16 763 (66.0)	5664 (33.8)	3449 (20.6)
Arab	6006 (23.6)	2383 (39.7)	1325 (22.1)
Ultra-Orthodox Jewish	2634 (10.4)	832 (31.6)	492 (18.7)
Birth year			
2000-2010	9979 (39.3)	2487 (24.9)	1291 (12.9)
2011-2020	15 424 (60.7)	6392 (41.4)	3975 (25.8)

Abbreviations: CMA, chromosomal microarray analysis; ID/GDD, intellectual disability or global developmental delay; NDD, neurodevelopmental disorder.

^a^ Percentages are by row.

^b^ Includes patients with autism, ID/GDD, and multiple diagnoses.

The largest NDD subgroup included patients with autism (10 311 [40.6%]), followed by epilepsy (5632 [22.2%]), ID/GDD (4393 [17.3%]), and cerebral palsy (1062 [4.2%]). In addition, 4005 patients (15.8%) had more than 1 of these diagnostic categories (multiple diagnoses) (Table; Figure 1B).

Overall, 49.0% of the cohort had autism, either as a single diagnosis (10 311 patients) or as part of multiple NDDs (2146 patients) (eTable 2 in Supplement 1), accounting for 0.52% of the total insured population born between 2000 and 2020. The number of patients diagnosed with major NDDs increased over time, peaking at 2226 patients born in 2014 (1.75% of that birth cohort) (Figure 1C), including 1.01% diagnosed with autism.

### Genetic Testing Use for NDDs

**Figure 2.**
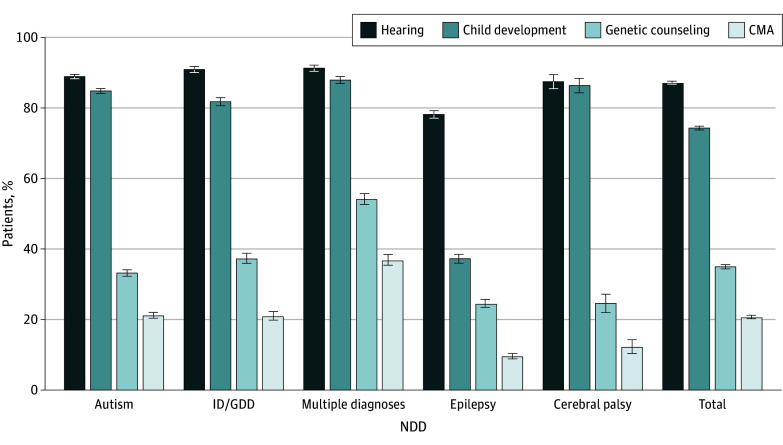
Clinical Interventions of Patients With Neurodevelopmental Disorders (NDDs) Error bars indicate 95% CIs. CMA indicates chromosomal microarray analysis; ID/GDD, intellectual disability or global developmental delay.

Intervention for children diagnosed with NDDs includes various components, such as developmental assessments and therapies, hearing examinations, and genetic evaluations; the latter comprises genetic counseling followed by genetic testing. Our data show that most patients diagnosed with major NDDs underwent hearing evaluations and developmental care (87.2% and 74.3%, respectively) (Figure 2). However, significantly fewer received genetic counseling (8879 patients [35.0%]; *z* = 89.09; *P* < .001), and even fewer underwent CMA testing (5265 patients [20.7%]; *z* = 120.79; *P* < .001) (Table). This finding suggests that approximately one-third of patients counseled did not proceed with CMA testing.

We focused on autism, ID/GDD, and multiple diagnoses, conditions for which genetic testing has been recommended by international guidelines for more than a decade.^23,36,37,38^ Even among this indicated group, only 38.7% of patients (7233 of 18 709) underwent genetic counseling (Table). This rate differed across diagnoses, ranging from 2169 of 4005 patients with multiple diagnoses (54.2%) to 3422 of 10 311 patients (33.2%) with autism alone, the largest group in the cohort (*z* = 23.08; *P* < .001) (Table; Figure 2). The overall CMA testing rate in this subset was 24.5% (4592 of 18 709 patients) (63.5% of patients counseled), with significantly higher rates in those with multiple diagnoses (1478 of 4005 [36.9%]) compared with those with ID/GDD (925 of 4393 [21.1%]) (*z* = 16.04; *P* < .001) and autism (2189 of 10 311 [21.2%]) (*z* = 19.28; *P* < .001). No significant difference was observed between ID/GDD and autism.

Genetic counseling for patients diagnosed with epilepsy or cerebral palsy was the lowest (1385 of 5632 [24.6%] and 261 of 1062 [24.6%], respectively) (Table; Figure 2). Even among patients with multiple NDDs, the rate of patients with cerebral palsy who underwent genetic evaluation was significantly less compared with patients with multiple diagnoses without cerebral palsy (genetic counseling: 41.7% vs 59.9%; *z* = −10.71; CMA: 26.6% vs 41.7%; *z* = −9.21) (both *P* < .001) (eFigure 1 in Supplement 1).

### Genetic Counseling and Testing in Different Subpopulations

We analyzed demographic factors to assess their association with genetic evaluation. Rates of genetic testing showed a small difference between male and female patients (21.2% vs 19.6%; *P* = .003) (Table). Patients born after 2010 were significantly more likely to undergo genetic evaluations compared with those born before 2010, with CMA testing rates of 25.8% vs 12.9% (*P* < .001).

**Figure 3.**
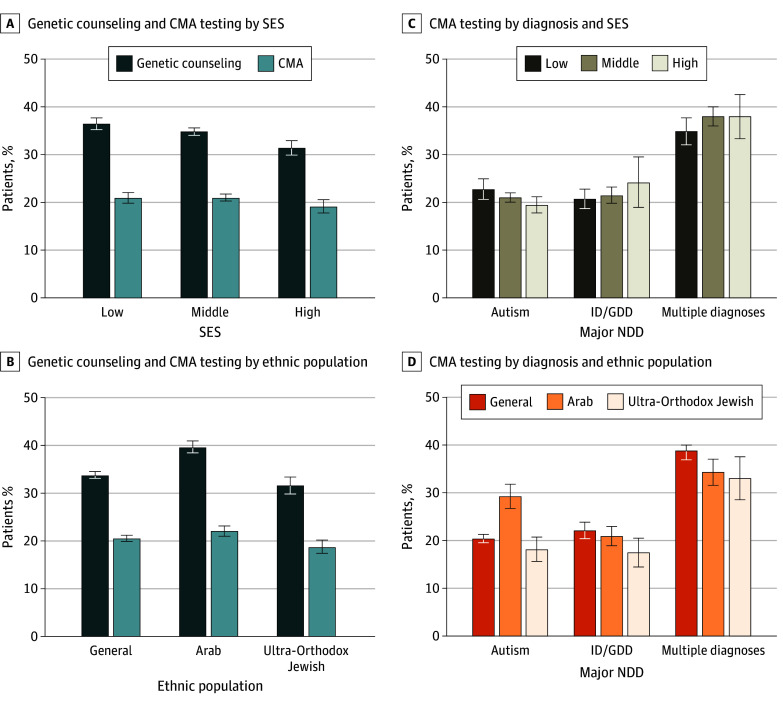
Use of Genetic Counseling and Chromosomal Microarray Analysis (CMA) Error bars indicate 95% CIs. ID/GDD indicates intellectual disability or global developmental delay; NDD, neurodevelopmental disorder; SES, socioeconomic status.

We analyzed sociodemographic factors, including SES and minority (Arab and Ultra-Orthodox Jewish) groups. Genetic counseling rates across these groups ranged from 31.4% in patients of high SES to 39.7% in Arab patients (Table; Figure 3A-B; eFigure 2 in Supplement 1). Chromosomal microarray analysis testing rates ranged from 18.7% in Ultra-Orthodox Jewish patients to 22.1% in Arab patients, with per-diagnosis analysis showing comparable trends (Figure 3C-D; eFigure 2 in Supplement 1). These results show that the overall underuse of genetic evaluations was relatively consistent across all subpopulations.

### Representation of Patients With NDDs From Low SES and Minority Groups

**Figure 4.**
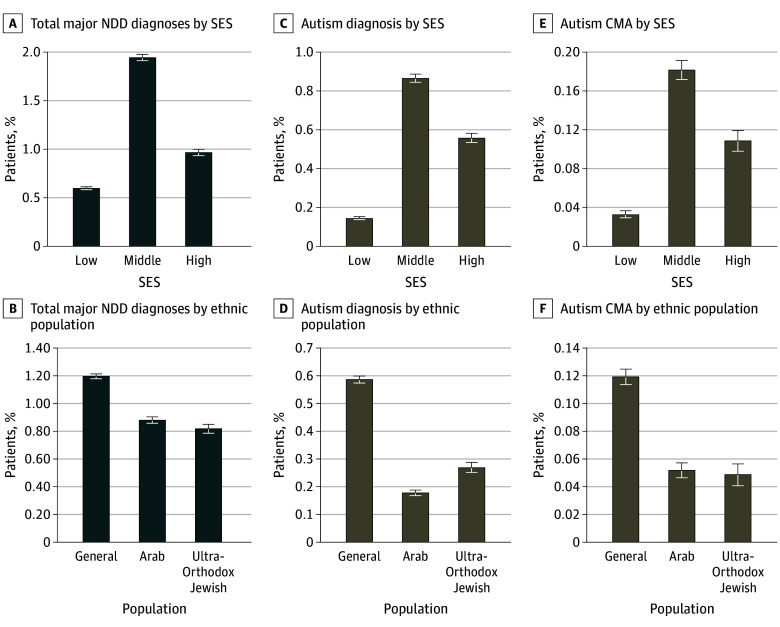
Demographic Characteristics of Patients With Any Major Neurodevelopmental Disorder (NDD) and With Autism Only Major NDD diagnoses included autism, intellectual disability or global developmental delay, or multiple diagnoses. Error bars indicated 95% CIs. CMA indicates chromosomal microarray analysis; SES, socioeconomic status.

We examined whether disparities in initial NDD diagnoses at the population level contributed to differences in genetic testing rates. Patients of low SES accounted for 41.4% of the overall population but only 23.4% of the NDD cohort (5948 of 25 403 patients; *z* = −57.83; *P* < .001) (Table; eTables 3 and 4 in Supplement 1). Similarly, Arab and Ultra-Orthodox Jewish patients, who made up 28.4% and 13.4% of the total population, respectively, were underrepresented in the major NDD cohort (Arab patients: 23.6%; *z* = −16.54; Ultra-Orthodox Jewish patients: 10.4%; *z* = −14.01) (both *P* < .001) (Table), suggesting that NDDs may be underdiagnosed in low SES and minority populations (Figure 4A-B).

Per-diagnosis analysis revealed that the disparity in NDD diagnoses was primarily associated with the underdiagnosis of autism in low SES and minority groups (Figure 4C-D). Autism diagnosis rates varied significantly across SES groups (0.14% in the low SES group [1.4 per 1000] vs 0.87% [8.7 per 1000] and 0.56% [5.6 per 1000] in the middle and high SES groups) (all *P* < .001) (Figure 4C). Similarly, minority groups had significantly lower autism diagnosis rates (0.18% for Arab patients and 0.27% for Ultra-Orthodox Jewish patients) compared with the general population (0.59%) (all *P* < .001) (Figure 4D), suggesting a relative underdiagnosis of 54% to 83%. The differences in diagnosis rates for other NDDs across ethnic groups were less pronounced and are detailed in eFigure 3 in Supplement 1.

The combination of low NDD diagnosis rates with the overall underuse of genetic evaluation contributed to 58% to 83% lower absolute rates of CMA testing at the population level in low-resource populations. For example, 0.03% of patients with low SES were tested compared with 0.18% with middle SES (*P* < .001) (Figure 4E). Similarly, 0.05% of Arab patients and 0.05% of ultra-Orthodox Jewish patients were tested compared with 0.12% in the general population (both *P* < .001) (Figure 4F). Lower rates of autism diagnosis and genetic testing were observed in peripheral regions of Israel compared with the more economically prosperous central region (eFigures 4 and 5 in Supplement 1). These findings align with the overrepresentation of Arab and Ultra-Orthodox Jewish patients among low SES populations in the peripheral regions.

## Discussion

In this cohort study of individuals with major NDDs born between 2000 and 2020 and followed up through 2023, we examined rates of genetic counseling and CMA. This period marked the integration of CMA as a first-line test for autism and ID/GDD.^4,22,23,24,38^ More powerful next-generation sequencing technologies had not yet been routinely integrated into Israel’s national health system, making CMA the primary indicator of genetic testing use over these decades.

This population-based study is the first in our knowledge to expand on prior smaller cohort research. Our study revealed that genetic counseling and CMA were underused, with only 35.0% of individuals diagnosed with major NDDs receiving counseling and 20.7% undergoing CMA testing. Even among patients with clear recommendations (those with autism, ID/GDD, multiple diagnoses), just 38.7% received counseling and 24.5% completed testing. Other medical management practices, such as developmental care and hearing evaluations, were conducted at much higher rates, indicating that patients were under routine follow-up. Lower rates of developmental follow-up in epilepsy may reflect the presence of benign forms not associated with additional neurodevelopmental issues.^39^ Yet, genetic services remained underused despite coverage by Israel’s national health insurance.

Barriers may include low referral rates and limited awareness of the benefits among parents and health care professionals.^19,40,41,42,43^ Additionally, bureaucratic processes and long wait times for genetic consultations can make access to these services more difficult.^44,45^ Some families may also be concerned about privacy and trust.^46^ In Israel, additional challenges include religious considerations, as Orthodox Jewish and Islamic traditions may also influence testing uptake.^47,48,49,50,51,52^

Of note, less than two-thirds of patients underwent CMA testing despite genetic counseling and coverage by Israel’s national health insurance. This dropout was consistent across all diagnoses and populations, suggesting that the process itself may deter families from completing it.

Clinical diagnosis was associated with undergoing genetic evaluation, with the highest rates among patients with multiple diagnoses, which may have been due to greater awareness. The utility of genetic testing in cerebral palsy and epilepsy has only recently been recognized,^13,53^ and CMA is not always a first-line test. Accordingly, our study found the lowest genetic counseling rates in cerebral palsy and epilepsy at 24.6% each. Among patients with cerebral palsy and additional NDD diagnoses, counseling and testing rates were lower than in other combinations, reflecting historical assumptions about nongenetic etiologies (eg, birth complications). As the field advances, updated guidelines and policies for cerebral palsy and epilepsy may become crucial for optimal patient care.

Rates of genetic evaluation have improved over time, with patients born after 2010 being more likely to be tested. The apparent decline in diagnoses among children born in later years may reflect shorter follow-up periods during which formal diagnoses, such as ID, which is made at later developmental stages, may not yet have been established. However, most patients born earlier, when genetic testing was not routinely offered, remain unevaluated, representing an important cohort for genetic evaluation as they, as well as their siblings, approach reproductive age.

Detailed demographic analysis showed no major differences in genetic evaluation rates based on SES, minority population (Arab and Ultra-Orthodox Jewish), or geographic location among patients diagnosed with NDDs. Since the underuse and high dropout rates were not associated with diagnosis or demographics, efforts should focus on raising medical team awareness to improve access, especially for individuals of reproductive age and those with cerebral palsy or epilepsy. Increasing testing completion from just 25% to a benchmark of 60%, a reasonable threshold in a quality-improvement program,^54^ could substantially raise definitive diagnoses.

Simplifying referrals, reducing wait times, and integrating testing into outpatient clinics may lower dropout rates. Including genetic evaluations in national quality-improvement programs could further incentivize testing.

Finally, autism diagnosis rates were significantly lower (as much as 54%-83% lower) among low SES and minority populations. In Israel, an autism diagnosis grants access to vital services and disability benefits, making disparities in diagnosis a barrier to essential resources. Raising awareness, especially in underserved populations, is key to ensuring equitable access to care.

### Strengths and Limitations

This study had several strengths, including a comprehensive data source and large cohort, the relative stability of the Israeli health care population, and a well-documented cohort to provide a robust foundation for identifying trends and barriers in genetic evaluation for NDDs. While the study provides valuable insights into the use of genetic evaluations for NDDs at the population level, it has several limitations. First, the analysis was conducted in CHS, which insures 52% of the Israeli population and, thus, may limit the generalizability of the findings to other health care systems. However, given Israel’s centrally regulated health care policies and similarities in health care infrastructure across many high-resource countries, these findings may reflect broader trends. Second, as with any population-based study, reliance on diagnostic codes may have introduced inaccuracies, including nonspecific entries. In addition, some individuals may have been lost to follow-up, potentially affecting data completeness. However, in-depth analysis indicated that such cases were infrequent and did not materially influence the main conclusions. Third, individuals born in the latter years of the study period (particularly after 2015) may not yet have reached the typical age for full diagnostic and genetic evaluation, limiting comparability with older individuals. In addition, this study focused on CMA as the primary genetic test, as it was the first-line diagnostic tool used during the study period. However, the analysis did not include data on next-generation sequencing techniques or individual test results due to privacy regulations. Future studies should aim to incorporate next-generation sequencing data and expand the geographic scope to validate these findings in other health care systems.

## Conclusions

While genetic testing is now integral to diagnosing and managing NDDs, this cohort study suggests that considerable gaps in use remain. Our findings from Israel align with trends observed in other high-resource countries, emphasizing the need for collaborative efforts by health care professionals, policymakers, and researchers. Addressing these gaps could enhance the quality of care for individuals with NDDs and their families, ensuring equitable access to genetic services worldwide.

## Notes

Supplement 1. **eTable 1.** All NDD Groups of the Original Cohort **eTable 2.** Number of Patients With Multiple Major NDDs **eTable 3.** Sociodemographic Details of the Study Population **eTable 4.** Sociodemographic Details of Clalit Health Services Insured Population **eFigure 1.** Genetic Counseling and Chromosomal Microarray Analysis (CMA) Performance in Different Cerebral Palsy (CP) Groups **eFigure 2.** Rate of Genetic Counseling (GC) and Chromosomal Microarray Analysis (CMA) Testing Among Different Diagnoses and Populations **eFigure 3.** NDD Diagnosis Rate for Subpopulations **eFigure 4.** Rate of Genetic Counseling (GC) and CMA Testing in Different Geographic Districts in Israel **eFigure 5.** NDD Diagnosis Rate by Geographic District Showing Total NDD Diagnoses and Autism Diagnoses

Supplement 2.
Data Sharing Statement
