# Supplementary material for: Genetic Testing of Neurodevelopmental Disorders in Israel
Source: JAMA Netw Open. 2025 Aug 19;8(8):e2527464. doi: 10.1001/jamanetworkopen.2025.27464 (PMC12365703; doi:10.1001/jamanetworkopen.2025.27464)
Supplement: Supplement 1. — eTable 1. All NDD Groups of the Original Cohort eTable 2. Number of Patients With Multiple Major NDDs eTable 3. Sociodemographic Details of the Study Population eTable 4. Sociodemographic Details of Clalit Health Services Insured Population eFigure 1. Genetic Counseling and Chromosomal Microarray Analysis (CMA) Performance in Different Cerebral Palsy (CP) Groups eFigure 2. Rate of Genetic Counseling (GC) and Chromosomal Microarray Analysis (CMA) Testing Among Different Diagnoses and Populations eFigure 3. NDD Diagnosis Rate for Subpopulations eFigure 4. Rate of Genetic Counseling (GC) and CMA Testing in Different Geographic Districts in Israel eFigure 5. NDD Diagnosis Rate by Geographic District Showing Total NDD Diagnoses and Autism Diagnoses [file jamanetwopen-e2527464-s001.pdf]

Supplemental Online Content

May D, Barshir R, Shahar M, Rose AJ, Shmueli D. Genetic testing of neurodevelopmental disorders in Israel. *JAMA Netw Open*. 2025;8(8):e2527464. doi:10.1001/jamanetworkopen.2025.27464

eTable 1. All NDD Groups of the Original Cohort

eTable 2. Number of Patients With Multiple Major NDDs

eTable 3. Sociodemographic Details of the Study Population

eTable 4. Sociodemographic Details of Clalit Health Services Insured Population

eFigure 1. Genetic Counseling and Chromosomal Microarray Analysis (CMA) Performance in Different Cerebral Palsy (CP) Groups

eFigure 2. Rate of Genetic Counseling (GC) and Chromosomal Microarray Analysis (CMA) Testing Among Different Diagnoses and Populations

eFigure 3. NDD Diagnosis Rate for Subpopulations

eFigure 4. Rate of Genetic Counseling (GC) and CMA Testing in Different Geographic Districts in Israel

eFigure 5. NDD Diagnosis Rate by Geographic District Showing Total NDD Diagnoses and Autism Diagnoses

This supplemental material has been provided by the authors to give readers additional information about their work.

eTable 1. All NDD Groups of the Original Cohort

Diagnosis	ICD10	Diagnosis group
Pervasive developmental disorders	F84	Autism Spectrum Disorder
Atypical autism	F84.0	Autism Spectrum Disorder
Autism spectrum disorder	F84.0	Autism Spectrum Disorder
Childhood autism	F84.0	Autism Spectrum Disorder
Infantile autism	F84.0	Autism Spectrum Disorder
Asperger's syndrome	F84.5	Autism Spectrum Disorder
Autism syndrome	F84.5	Autism Spectrum Disorder
Other pervasive developmental disorders	F84.8	Autism Spectrum Disorder
Pervasive developmental disorder nos	F84.9	Autism Spectrum Disorder
Pervasive developmental disorder unspecified	F84.9	Autism Spectrum Disorder
Spastic diplegia	G80.0	Cerebral Palsy
Infantile cerebral palsy unspecified	G80.9	Cerebral Palsy
Hemiplegia	G81	Cerebral Palsy
Hemiparesis	G81.9	Cerebral Palsy
Paraplegia	G82.20	Cerebral Palsy
Quadriplegia	G82.50	Cerebral Palsy
Cerebral palsy	Unknown	Cerebral Palsy
Epilepsy	G40	Epilepsy
Grand mal status	G40.301	Epilepsy
Continuous spike wave during slow wave sleep	G40.4	Epilepsy
Absence	G40.A01	Epilepsy
Absence (petit mal) status	G40.A01	Epilepsy
Benign neonatal familial convulsions	P90	Epilepsy
Generalized epilepsy with febrile seizures plus	R56.01	Epilepsy
Benign myoclonic epilepsy of infancy	R56.9	Epilepsy
Convulsions/seizures	Unknown	Epilepsy
Epilepsy; all types	Unknown	Epilepsy
Multifocal epilepsy	Unknown	Epilepsy
Mild mental retardation	F70	ID/GDD
Mild mental retardation no/minimal impairment of behaviour	F70	ID/GDD
Mild mental retardation other impairments of behaviour	F70	ID/GDD
Mild mental retardation significant impairment of behaviour	F70	ID/GDD
Moderate mental retardation	F71	ID/GDD
Moderate mental retardation other impairments of behaviour	F71	ID/GDD
Moderate mental retardation significant impairment of behav.	F71	ID/GDD
Severe mental retardation	F72	ID/GDD
Severe mental retardation no/minimal impairment of behav.	F72	ID/GDD
Profound mental retardation significant impairment of behav.	F73	ID/GDD
Other mental retardation	F78	ID/GDD
Other mental retardation significant impairment of behaviour	F78	ID/GDD
Global development delay	Unknown	ID/GDD
Mental retardation (incl. Down)	Unknown	ID/GDD
Retardation mental	Unknown	ID/GDD
Attention deficit disorder with hyperactivity	F90.1	ADHD
Specific developmental disorder of motor function	F82	Delayed Motor Milestones
Delayed motor milestones	Unknown	Delayed Motor Milestones
Mixed developmental disorder	F82	Mixed Developmental Disorder
Mixed specific developmental disorders	Unknown	Mixed Developmental Disorder

© 2025 May D et al. *JAMA Network Open*.

eTable 2. Number of Patients With Multiple Major NDDs (by NDD combinations, total n=4,005).

**Diagnosis combination**	**Count**
ASD+ID/GDD	1324
Epilepsy+ID/GDD	717
CP+Epilepsy	510
ASD+Epilepsy	504
CP+Epilepsy+ID/GDD	317
CP+ID/GDD	315
ASD+Epilepsy+ID/GDD	195
ASD+CP	53
ASD+CP+ID/GDD	27
ASD+CP+Epilepsy	24
ASD+CP+Epilepsy+ID/GDD	19

© 2025 May D et al. *JAMA Network Open*.

eTable 3. Sociodemographic Details of the Study Population

Diagnosis	(by socio-economic level)	n	% of diagnosis cohort
**Autism**	Low	1441	13.98%
	Middle	6400	62.07%
	High	2115	20.51%
	Unknown	355	3.44%
**ID/GDD**	Low	1533	34.90%
	Middle	2224	50.63%
	High	252	5.74%
	Unknown	384	8.74%
**Multiple diagnoses**	Low	1094	27.32%
	Middle	2239	55.91%
	High	416	10.39%
	Unknown	256	6.39%
**CP**	Low	339	31.92%
	Middle	500	47.08%
	High	118	11.11%
	Unknown	105	9.89%
**Epilepsy**	Low	1541	27.36%
	Middle	3002	53.30%
	High	753	13.37%
	Unknown	336	5.97%
**Diagnosis**	**(by sector)**	**n**	**% of diagnosis cohort**
**Autism**	General population	8228	79.80%
	Arabs	1214	11.77%
	Ultra-orthodox Jews	869	8.43%
**ID/GDD**	General population	2228	50.72%
	Arabs	1554	35.37%
	Ultra-orthodox Jews	611	13.91%
**Multiple diagnoses**	General population	2427	60.60%
	Arabs	1160	28.96%
	Ultra-orthodox Jews	418	10.44%
**CP**	General population	532	50.09%
	Arabs	406	38.23%
	Ultra-orthodox Jews	124	11.68%
**Epilepsy**	General population	3348	59.45%
	Arabs	1672	29.69%
	Ultra-orthodox Jews	612	10.87%

© 2025 May D et al. *JAMA Network Open*.

eTable 4. Sociodemographic Details of Clalit Health Services Insured Population

Population feature	Sub population	Count	% of total insured population
Socioeconomic level	Low	995,570	41.37%
	Middle	739,423	30.72%
	High	378,535	15.73%
	Unknown	293,235	12.18%
Sector	General population	1,402,548	58.28%
	Arabs	682,344	28.35%
	Ultra-orthodox Jews	321,871	13.37%

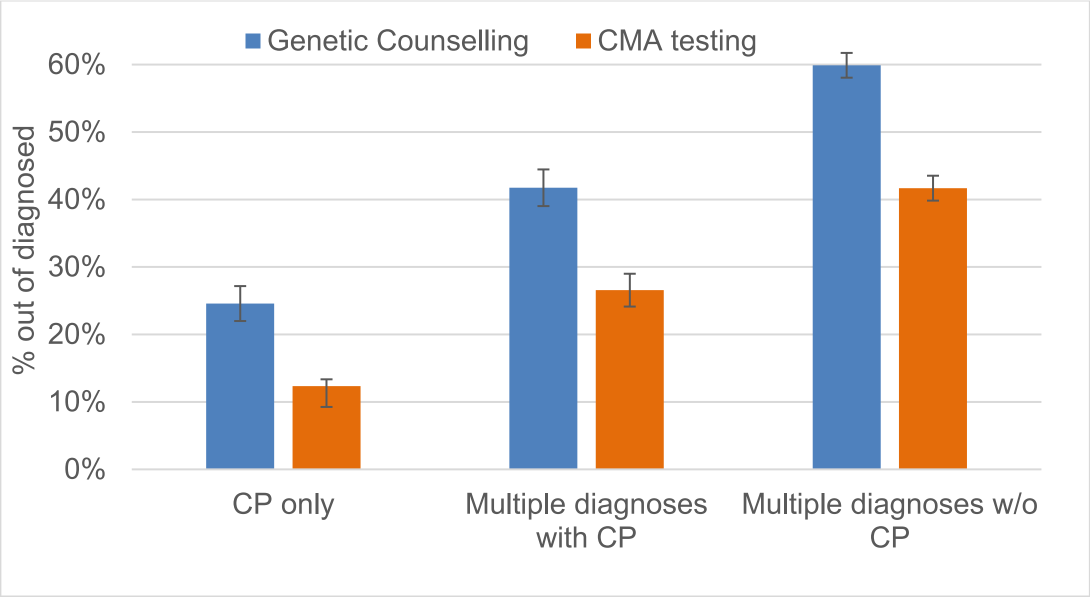

eFigure 1. Genetic Counseling and Chromosomal Microarray Analysis (CMA) Performance in Different Cerebral Palsy (CP) Groups. CP only: patients diagnosed with CP as a single diagnosis. multiple diagnoses with CP: patients with multiple diagnoses, including CP. Multiple diagnoses with-out (w/o) CP: patients with multiple diagnoses, but not CP.

© 2025 May D et al. *JAMA Network Open*.

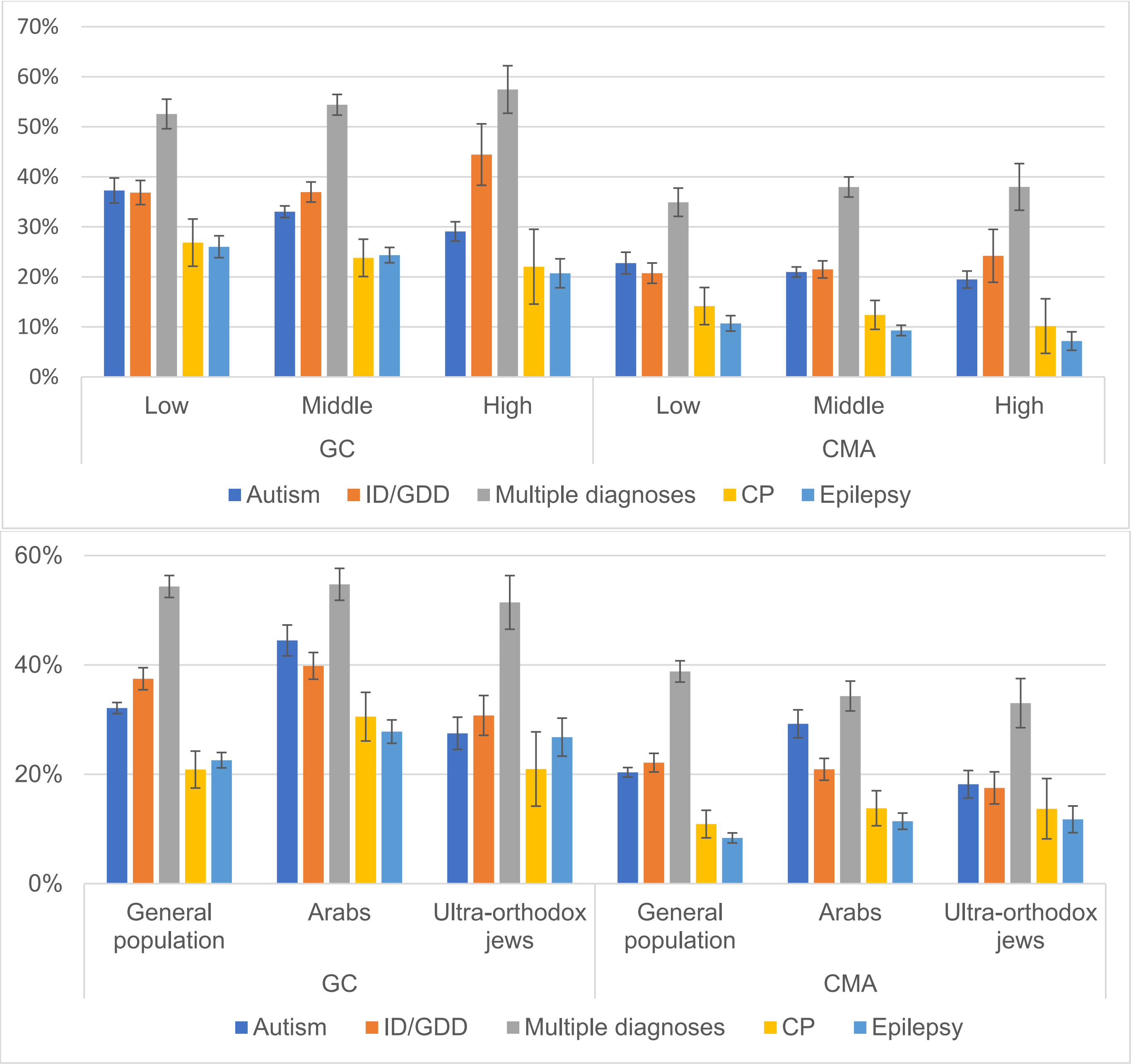

eFigure 2. Rate of Genetic Counseling (GC) and Chromosomal Microarray Analysis (CMA) Testing Among Different Diagnoses and Populations. Shown are percent diagnosed among low, middle, or high socioeconomic levels (top), or at the general population, or among Arabs and Ultra-orthodox Jews (bottom).

© 2025 May D et al. *JAMA Network Open*.

A

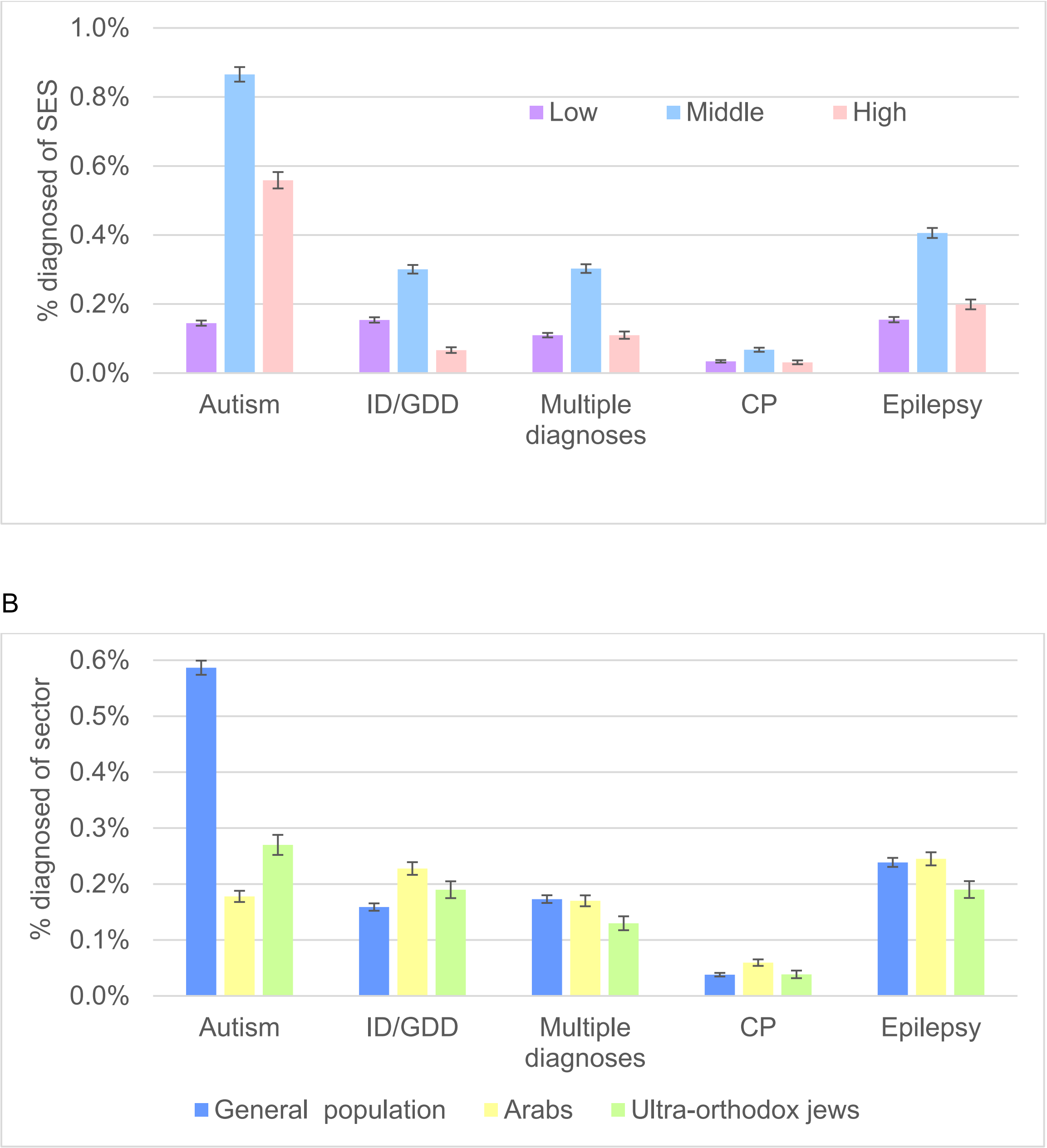

eFigure 3. NDD Diagnoses Rate for Subpopulations. (A) In different socioeconomic levels. p<0.001 for all diagnoses for all socioeconomic status (SES) comparison to middle SES, adjusted for multiple comparisons.

© 2025 May D et al. *JAMA Network Open*.

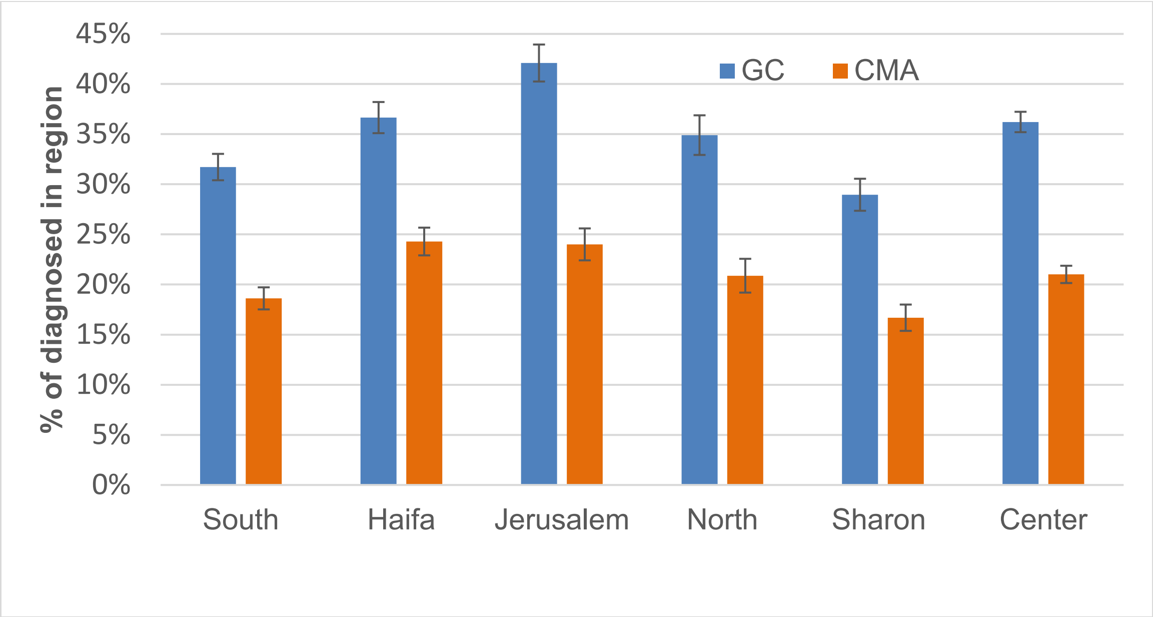

eFigure 4. Rate of Genetic Counseling (GC) and CMA Testing in Different Geographic Districts in Israel. Bar chart shows the percentage of children who underwent genetic counselling (orange) and CMA testing (blue) out of those diagnosed with NDD in each region, along with 95% confidence intervals.

© 2025 May D et al. *JAMA Network Open*.

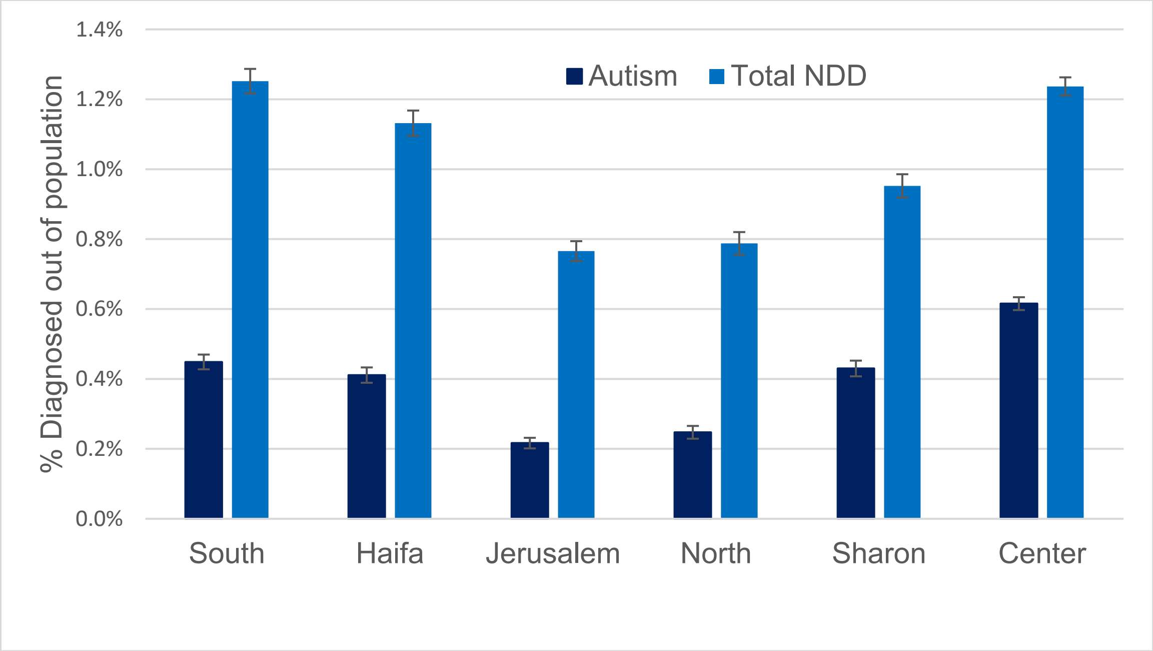

eFigure 5. NDD Diagnosis Rate by Geographic District Showing Total NDD Diagnoses and Autism Diagnoses. Bar chart shows the percentage of children diagnosed with autism and total NDD in each region, along with 95% confidence intervals. Autism diagnosis rates in all regions (South, Haifa, Jerusalem, North, and Sharon) were significantly lower compared to the Center region ($p < 0.001$ for all comparisons).

Total NDD diagnosis rates were also significantly lower in all regions compared to the Center (adjusted $p < 0.001$), except in the South, where rates were similar to those in the Center (adjusted $p = 0.49$).

© 2025 May D et al. *JAMA Network Open*.
