# Supplementary material for: Genetic Testing of Neurodevelopmental Disorders in Israel
Source: JAMA Netw Open. 2025 Aug 19;8(8):e2527464. doi: 10.1001/jamanetworkopen.2025.27464 (PMC12365703; doi:10.1001/jamanetworkopen.2025.27464)
Supplement: Supplement 2. — Data Sharing Statement [file jamanetwopen-e2527464-s002.pdf]

Data Sharing Statement

May. Genetic Testing of Neurodevelopmental Disorders in Israel. *JAMA Netw Open*. Published August 19, 2025. doi:10.1001/jamanetworkopen.2025.27464

Data
Data available: Yes
Data types: Other (please specify)
Additional Information: Data summary that supports the findings of this study is available on request from the authors. The data are not publicly available due to restrictions [e.g. their containing information that could compromise the privacy of research participants].
How to access data: Please contact the authors
When available: With publication

Supporting Documents
Document types: None

Additional Information
Who can access the data: anyone requesting the data
Types of analyses: for research purpose
Mechanisms of data availability: With investigator support
